# Web-Supported Social Network Testing for HIV Among Men Who Have Sex With Men With a Migration Background: Protocol for a Mixed Methods Pilot Study

**DOI:** 10.2196/14743

**Published:** 2020-02-10

**Authors:** Eline Op de Coul, Chantal den Daas, Ralph Spijker, Titia Heijman, Marvin de Vos, Hannelore Götz, Koenraad Vermey, Wim Zuilhof, Jossy Van den Boogaard, Udi Davidovich, Freke Zuure, PREVENT Collaborators

**Affiliations:** 1 Centre for Infectious Disease Control National Institute for Public Health and the Environment Bilthoven Netherlands; 2 Interdisciplinary Social Science Utrecht University Utrecht Netherlands; 3 Aidsfonds–Soa Aids Nederland Amsterdam Netherlands; 4 Public Health Service of Amsterdam Amsterdam Netherlands; 5 Public Health Service Rotterdam-Rijnmond Rotterdam Netherlands; 6 Department of Social Psychology University of Amsterdam Amsterdam Netherlands; 7 See Authors' Contributions

**Keywords:** HIV, social network testing (SNT), men who have sex with men (MSM), community-based testing, HIV self-testing

## Abstract

**Background:**

Of newly diagnosed HIV positive men who have sex with men (MSM) in the Netherlands, 29% have a non-Western migration background (MSM-NW). Among MSM-NW, HIV positivity rates are high (0.8%-2.0%), as is the proportion of late stage infections (39%). Factors such as HIV and sexual orientation–related stigma may form barriers for timely testing. Innovative approaches for HIV testing are needed to better reach MSM-NW. Social network testing (SNT) for HIV is an evidence-supported approach where peer recruiters identify persons (network associates) who could benefit from testing in their social or sexual networks. Web-supported SNT might be particularly promising for reaching people who may not be reached by regular care.

**Objective:**

The purpose of this paper is to describe the design of our pilot PREVENT (Peer-Empowered Voluntary Extended Network Testing). In this pilot, we will explore whether SNT using HIV self-tests is feasible and acceptable among MSM-NW in the Netherlands and whether it reaches those who were never or not recently tested for HIV (>1 year ago).

**Methods:**

The project aims to include 50 to 60 MSM and MSM-NW peers who will distribute 4 to 5 oral HIV self-tests each aiming to reach 200 network associates (NAs). Enrollment of peers includes 4 steps: (1) fostering interest in becoming a peer by health care professionals at sexual health clinics, HIV treatment clinics, and community settings; (2) sending peer contact information to the peer coordinator; (3) registering peers and giving program instructions by the peer coordinator and referring to the Web-based training at time2test; and (4) receiving precoded HIV self-tests for distribution in the peers’ networks. NAs who receive the self-test will log in with their test package code in the time2test application for step-by-step test instructions. After testing is complete, NAs receive tailored follow-up information depending on their test result.

**Results:**

Between January and May 2019, 10 STI clinics and 7 HIV treatment clinics started recruiting peers. Results of the PREVENT pilot are expected in December 2020.

**Conclusions:**

This is the first Web-supported peer-driven SNT pilot using HIV self-tests in the Netherlands and one of the first in Europe. Implementation is considered successful if it reaches MSM-NW who were never or not recently tested for HIV. Additionally, it may encourage conversations within the networks about risk behavior and barriers to HIV testing, potentially contributing to the Joint United Nations Programme on HIV/AIDS goal of zero HIV infections.

**Trial Registration:**

Netherlands Trial Registry NL7424; https://www.trialregister.nl/trial/7424

**International Registered Report Identifier (IRRID):**

DERR1-10.2196/14743

## Introduction

People who are unaware of their HIV infection are more likely to be a source of HIV infection for others and are unable to benefit from (early) treatment [1,2]. Of HIV prevention interventions evaluated to date, increased HIV testing combined with early treatment resulting in viral suppression has been shown to have the most substantial effect on HIV transmission at population level [3]. In the Netherlands, men who have sex with men (MSM) account for the majority (68%) of new HIV infections. Of those, 29% have a non-Western migration background (MSM-NW). Among MSM-NW, HIV positivity rates are fairly high (0.8-2.0%) [4] with a high proportion (39%) of late stage infections (CD4 <350 cells/mm^3^) [4-6]. High proportions of late stage infections may indicate that MSM-NW are not well reached by regular, provider-based test facilities. Innovative testing approaches not dependent on patient- or provider-initiated testing are needed to reach MSM-NW for HIV testing.

Peer-driven HIV testing in social networks is an evidence-supported approach to improve HIV testing rates in difficult-to-reach populations [7-9]. These social network testing (SNT) approaches can reach hidden individuals at high risk for HIV infection and those who are unaware of their HIV status, as shown in various studies mostly in the United States [10-14]. SNT is based on the concept that individuals with similar characteristics (sociocultural and behavioral) are linked together to form social networks with similar sexual (risk) behavior [9,15]. Persons within a network may encounter similar sexual situations or may influence each other’s behavior. Some studies even showed that network-level variables were stronger predictors of risk behavior outcomes than individual-level variables [16]. Among networks in which HIV prevalence and sexual risk behavior are high, this interconnectedness between people can be used to roll out an intervention aimed at increasing testing uptake and locating people with undiagnosed HIV infection. When SNT is combined with HIV self-tests, this might overcome barriers to provider-based testing, such as fear of being seen at a clinic by people they know or reluctance to disclose their sexual identity to a health care professional [17-19]. Although several SNT studies for HIV testing in MSM networks have been published, it has not been attempted to great extent in combination with HIV self-tests and Web-based support. The HIV self-test with Web-based support enables the network associate (NA) to test anonymously in their home environment or another safe place, using step-by-step instructions, videos, and information tailored to their test result.

The main goal of our pilot, PREVENT (Peer-Empowered Voluntary Extended Network Testing), is to investigate whether SNT [8,10,20] is feasible and acceptable in MSM-NW networks in the Netherlands. The major target group is MSM with a non-Western migration background (eg, Central and Eastern Europe, Africa, Central and South America, and Asia). Transgender people are also invited to participate as they often encounter similar barriers to testing. Transgender female sex workers are at especially high risk for HIV infection and often exposed to physical and sexual violence [21,22].

PREVENT will focus specifically on the feasibility and acceptability of SNT using HIV self-tests. If we can recruit and support high-risk peers—HIV-positive or HIV-negative—to distribute self-tests among their social contacts, we can decrease rates of people never being tested, detect undiagnosed HIV infections earlier, and learn how SNT can be improved. Furthermore, the self-test could act as an object connecting people and improve conversations and openness about HIV.

PREVENT was developed as a blended intervention, combining face-to-face and Web-based strategies. It will include learning strategies for recruitment of instructing and motivating peers and NAs for SNT with HIV self-tests. Using a Web-based application for instructions and support for both peers and NAs has several advantages for SNT among MSM. Internet use is high for meeting sex partners or seeking health information [23], and the internet is available 24/7, allows anonymous participation, and enables the provision of tailored information and linkage with other Web-based sexually transmitted infection (STI) testing services.

This pilot intends to explore and understand HIV testing through social networks of MSM and MSM-NW with the primary aim being to study feasibility and acceptability of SNT using HIV self-tests. Secondary aims are to assess the effectiveness of SNT regarding diagnosing HIV and explore the effect of SNT on openness toward HIV and HIV testing.

## Methods

### Recruitment of Peers

MSM and MSM-NW will be recruited for the PREVENT pilot at 10 STI clinics and 7 HIV treatment clinics throughout the country. In the Netherlands, 24 STI clinics, mostly within public health services, provide free-of-charge STI/HIV testing and care for high-risk groups; 30% of all consultations are among MSM, with 45,553 consultations in 2017 [4]. In 2017 at these clinics, HIV positivity rates among Western MSM were 0.5% and 1.2% for non-Western MSM, with the highest percentages for MSM originating from Latin America (2.0%) and Eastern Europe (1.8%). Among transgender people, HIV positivity was 1.4% [4]. People newly diagnosed with HIV are referred to one of the 26 HIV treatment centers [6]. Additionally, recruitment of peers will take place at community settings for lesbian, gay, bisexual, transgender, and intersex (LGBTI) persons, where HIV testing, hepatitis B vaccinations, and pre-exposure prophylaxis (PrEP) information are being offered by health care professionals or trained volunteers.

Recruitment of peers is accomplished in a stepwise process. MSM and MSM-NW who visit any of the recruitment sites are informed about the project and receive an information flyer. Inclusion criteria for peers include being aged 18 years or older, having a social network that includes MSM-NW, and being willing to distribute HIV self-tests to their NAs. A social network includes personal connections such as friends, acquaintances, (ex-)partners, sexual contacts, colleagues, etc. The size of a persons’ network is not an inclusion criterion. Non-Western is defined as first or second generation Caribbean, African, Eastern/Central European, Asian, or Central/Latin American. MSM from Western Europe who have a network of MSM-NW can also participate as peers, as can transgender individuals.

Persons who are interested in participating are asked to provide an email address or telephone number to the health professional, who then sends the contact details to the peer coordinator. Potential peers who received the information flyer can also register themselves via the project website [24]. Subsequently, peers are contacted by the peer coordinator who provides them with project information and registers them in the Web-based time2test application. The application sends a website link for creating a peer account. After completing the informed consent and baseline questionnaire on their account page, peers follow a short training (e-learning tool of 20 minutes) to prepare them for SNT testing. After completion of the e-learning tool, participants indicate they will sign up to the program by clicking on a button “I sign up as a peer” or they click on “I changed my mind and do not sign up as a peer.” If they sign up, they will receive a message telling them they will be contacted by the peer coordinator and will receive 5 precoded HIV self-tests by mail or at a nearby location (eg, closest STI clinic) for distribution in their social network.

### Ethics and Consent

The study was presented to the medical ethics committee of the Amsterdam University Medical Center (multicenter study reference number NL61922.018.17). The committee concluded that ethical approval was not needed as the Dutch Medical Research Involving Human Subjects Act does not apply for this study. The trial was registered with the Netherlands Trial Registry [NL7424]. All participants in PREVENT provide online informed consent.

### Sample Size

The primary objective of PREVENT is to assess the feasibility and acceptability of SNT among MSM-NW, and therefore a formal power analysis is not appropriate. However, we aim to enroll 200 NAs during a period of 12 to 18 months. To achieve this target, an estimated 50 to 60 peers who will distribute 4 to 5 test packages each should be recruited when they attend any of the participating sites. With these numbers, the pilot is expected to have 80% statistical power to observe differences of 15% in study outcomes (cross-sectional analyses) between subgroups, such as younger versus older age groups or by migration background and location of recruitment.

### Development of the E-Learning Tool for Peers

The e-learning tool is part of the time2test website that was developed for the PREVENT pilot. Findings of a qualitative prestudy among MSM-NW were used for website content and to design the e-learning tool [19]. This qualitative study, in which 13 MSM of various migration backgrounds recruited from STI clinics were interviewed, was based on the capability, opportunity, motivation, and behavior (COM-B) model and was focused on barriers and facilitators of SNT for HIV [25,26]. It included network characteristics (ie, who could benefit from SNT), peer skills to apply SNT successfully, assessments of sexual risks, abilities to motivate NAs to test, cultural or social aspects, and practical requirements for implementation. MSM-NW who were interviewed thought that SNT with HIV self-test was feasible and needed. They also indicated a need for support of peers in case of emotional reactions or a positive HIV diagnosis among their NAs. Openness and trust were considered important elements for successful implementation of SNT [19].

From the prestudy, learning objectives and delivery strategy were determined by the research team of PREVENT. Examples of learning objectives: peer can explain HIV transmission routes, peer can explain how the OraQuick HIV self-test (OraSure Technology, Inc) works, peer is motivated to prioritize friends within his inner circle of trust (eg, those never tested for HIV, having a migration background, and/or at sexual risk), peer is able to start a conversation about HIV testing. The learning objectives were translated into applications: the 6 steps of the e-learning tool in which they receive information and instruction videos on how to recruit NAs and motivate them to test. As there is no one-size-fits-all approach, example instruction videos were used. The prestudy showed that almost all men had an idea of how they would approach their contacts [19]. Peers are free (and probably know best how) to tailor their approach to suit their NAs.

### Content of the E-Learning Tool for Peers

**Figure 1:**
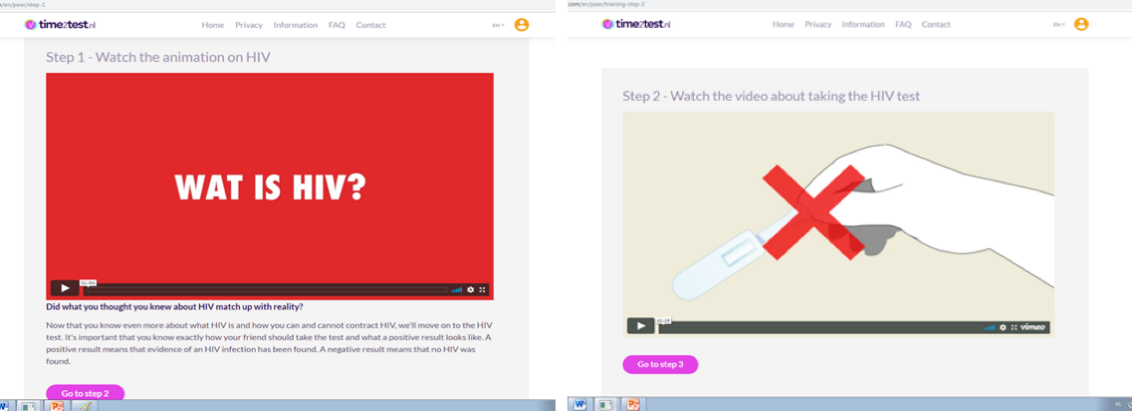
Screenshots of the time2test website showing animations on HIV (step 1 of e-tool, including an English voice over) and the oral HIV self-test (step 2).

**Figure 2:**
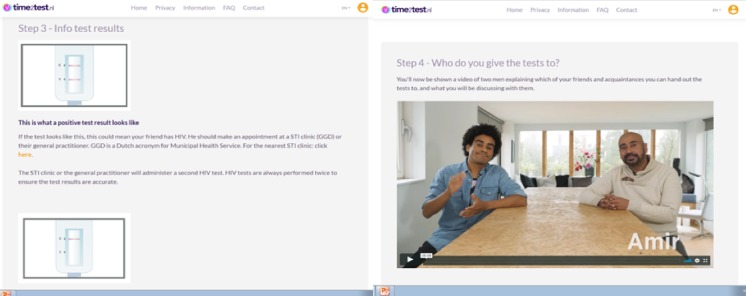
Screenshots of the time2test website showing the different test results (left) and a video explaining who to approach for testing (right).

**Figure 3:**
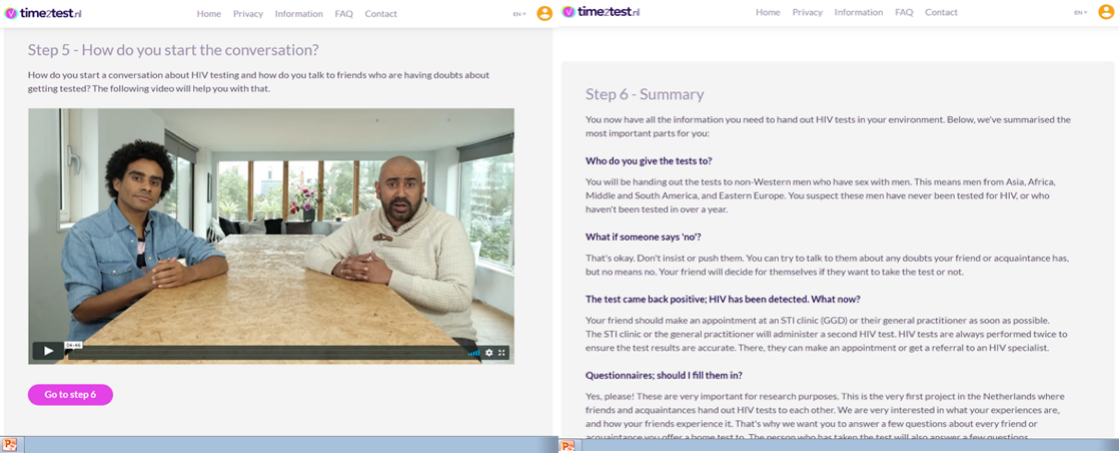
Screenshots of the time2test website showing a video on how to start a conversation about HIV self-testing (left) and a summary of information for peers (right).

In step 1, peers watch an animated video about HIV and how it is transmitted; in step 2, the use of the OraQuick HIV self-test is demonstrated (Figure 1). In step 3, the different test results of the oral HIV self-test are explained and illustrated by images of HIV positive, HIV negative, and failed test results. In step 4, peers watch a video in which two friends discuss how they selected their NAs for self-testing (Figure 2). It is explained that NAs are preferably unaware of their HIV status or not recently tested (>1 year ago). The video in step 5 discusses how they can start the conversation about HIV self-testing (Figure 3).

At the end of the e-learning tool, the peer has learned the following:

- Explain what HIV is and how it is transmitted
- Select NAs (friends, acquaintances, sex contacts, etc) for HIV self-testing
- Motivate NAs to test with an HIV self-test
- Guide NAs to the website for test instructions and counseling
- Deal with doubts or negative reactions of NAs about HIV testing
- Ask for support from the peer coordinator

### HIV Self-Testing for Network Associates

Network associates who accept the self-test from their peers log in to the time2test website (via a computer, tablet, or smartphone) with their test package number to create an anonymous test account based on a second code that is generated by the time2test application. The content of the time2test website as well as the instruction letters and manuals are provided in three languages (Dutch, English, and French).

**Figure 4:**
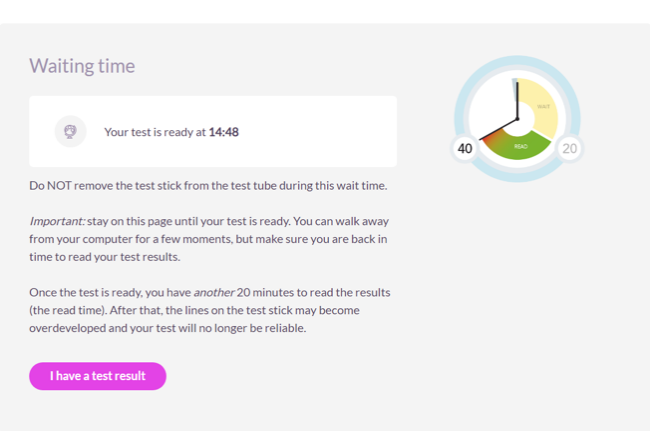
Image of one of the online testing steps for the oral HIV self-test.

After reading about the aims, terms of participation, and data privacy, they give online informed consent for participation by clicking on a button “I have read and understood the information above.” Next, they are taken to the baseline questionnaire (12 questions) on demographics, sexual orientation, HIV testing history, and sexual behavior. After completing the questionnaire, participants watch an animation about the OraQuick HIV self-test and go to the step-by-step test instructions. The HIV self-test uses oral fluid from the gums to detect antibodies against HIV-1 and HIV-2. In an OraQuick HIV self-test usability study on 900 individuals, sensitivity was reported as 99.4% (152 out of 153 positive individuals correctly identified their result as positive) and specificity as 99.0% (717 out of 724 negative individuals correctly identified their result as negative) [27]. The window phase of the test is 3 months. Both peers and NAs receive clear test instructions about this window phase and how to interpret the results. Test instructions have proven sufficient in an earlier study (HIVTest@Home trial) of the Amsterdam public health service where people could buy the self-test online [28]. NAs are instructed to check the content of the test package that includes an instruction letter, the self-test, and paper test manual. The paper manual contains similar information on testing and follow-up steps for NAs without internet access. After sample is collected and placed in the tube, the test result is directly readable after 20 minutes. The website contains a timer that shows when the test is ready (Figure 4). After the waiting time, images appear with the possible test results (1 line, 2 lines, or no lines) that NAs can compare with their own test. NAs have another 20 minutes to read the result. For each image, tailored information is provided that explains the test result and various posttest counseling options.

### Follow-Up

NAs with a reactive (HIV positive) test are informed about the importance of having a confirmation test and preventing (possible) further transmission of HIV. They are referred for confirmatory testing to an STI clinic in their region (telephone numbers provided through the STI clinic finder) where they are given priority, or they can go to their general practitioner (GP). They can print or download a referral letter on their phone for the health care professional. The referral letter explains what HIV self-test was used and the need for confirmation testing. They can also bring the test manual from their test package. When the confirmation test is HIV positive, they are directly referred to the nearest HIV treatment clinic as part of standard practice.

NAs with a nonreactive test are referred to the STI clinic, GP, or the Man tot Man (Testlab) website for repeated HIV testing after 3 months, testing for other STIs, and Web-based interventions aimed at reducing sexual risk behavior and increasing PrEP use.

In case the self-test failed, NAs can ask for a new test via their peer or they can send an email to the project mailbox that will be answered by the peer coordinator. NAs who have questions about the test procedure are referred to the Frequently Asked Questions page on the website or they can contact a health care professional by telephone through the STI clinic finder on the website.

### Data Collection and Study Outcomes

Peers and NAs provide online informed consent for participation and data collection directly after the online project information page and prior to any online data collection. The baseline questionnaires inquire about age, migration background, educational level, sexual behavior (NAs only), and HIV testing history (NAs only) (Textbox 1). After peers complete the training, they receive 5 test packages. For each person they approach with the self-test, they record a few baseline characteristics (type of contact, age, ethnicity, education, HIV testing history) on the website. If the self-test is accepted, peers activate the test package number by clicking on the number of the test (out of a list with 5 package numbers). The next time that an NA accepts a self-test, the 4 remaining package numbers will appear, and this process continues until no packages are left. If the self-test is not accepted, peers record a reason for nonacceptance. Peers are offered the opportunity to ask for additional self-tests in case they are able and willing to distribute more tests.

After their SNT period, peers will be invited to participate in an evaluation survey and/or qualitative interview about their experiences with SNT, their role as a peer, and their relationship with the NAs they approached. We will examine factors supporting the acceptance of the social network approach, barriers that peers experience during the recruitment process, and outcomes on communication about HIV testing (eg, Did offering the test affect the relationship between the peer and NA in any way?).

NAs who used the self-test are asked to provide a voluntary email address in case they are willing to be contacted for a follow-up questionnaire after 3 weeks or become a peer recruiter themselves. The follow-up questionnaire contains questions about their experiences with SNT and the Web-based service for self-testing. Not giving consent to these options does not affect the ability to participate in the project in any way.

The following outcomes will be examined (by study aim):

- Study feasibility and acceptability of SNT using HIV self-tests:
  - Numbers of registered peers, peers finishing the training, peers starting SNT
  - Peer profiles (eg, age, gender, ethnicity, area of residence, education, location recruited as peer)
  - Network index (accepted/used tests divided by the number of peers)
  - Numbers of NAs rejecting, accepting, and using the self-test
  - NA profiles (eg, age, gender, ethnicity, homo/bisexual, years living in the Netherlands, residence status, HIV testing history, number of sexual partners in the last 12 months)
  - Types of relationships between peers and NAs
  - Locations of self-test offer
- Assess the effectiveness of SNT regarding diagnosing HIV:
  - Number of newly diagnosed HIV infections (self-reported and/or confirmed at STI clinic)
  - Network yield (newly diagnosed HIV infections divided by the number of NAs/peers)
  - NA characteristics associated with newly diagnosed HIV infections
  - Peer characteristics associated with the ability to identify NAs with undiagnosed HIV infection
- Explore the effect of SNT on openness toward HIV and HIV testing:
  - Encouragement of conversations about risk behavior and HIV (testing) within networks
  - Reasons for nonacceptance of HIV self-test
  - Positive or negative reactions by NAs
  - Peer support

**Data collection among peers and network associates in the PREVENT pilot.**

Peers

- Inclusion:
  - Demographics (age, gender, ethnicity, residence, education level, location of recruitment)
  - Approval to participate as a peer
  - Approval for follow-up interview
- Follow-up:
  - Experiences with e-learning tool
  - Experiences with supervision by the peer-coordinator
  - Experiences with distibution of HIV self-tests
  - Reactions to the test offer
  - Shared test experience
  - Communication about HIV testing and sexuality
  - Willingness to distribute more self-tests

Approached network associates (completed by peers)

- Inclusion:
  - Demographics (age, gender, ethnicity, education level)
  - Sexual preference
  - Type of network contact
  - HIV testing history
  - HIV self-test acceptance and date of acceptance
  - Reason for nonacceptance and date of acceptance

Participating network associates

- Inclusion:
  - Demographics (age, gender, ethnicity, area of residence, education level, residential status, years in Netherlands)
  - Sexual behavior
  - HIV testing history
  - Approval for follow-up questionnaire

- Follow up:
  - Reason for acceptance of HIV self-test
  - Experience with the HIV self-test offer
  - Experience with conducting the HIV self-test
  - Would recommend the HIV self-test to others
  - HIV self-test conducted in presence of others
  - Location of conducting the HIV self-test
  - HIV test result
  - Location of confirmation test (HIV positive self-test only)
  - Result of confirmation test (HIV positive self-test only)
  - Visited HIV treatment clinic (if confirmed HIV positive)
  - Support by the peer
  - Sharing the test result with others
  - Communication about HIV in social network
  - Willingness to become a peer
  - Willingness to be interviewed
  - Changes over time in online visitors

Website tracking data

- Inclusion:
  - Frequency of log-ins
  - Number of webpages accessed

### Data Analyses

Descriptive analyses will be used to examine participation rates and characteristics of peers and NAs. The databases containing epidemiological data of peers and NAs can be linked based on the test package number. Therefore, we can identify person profiles (sexual risk, HIV testing history) as well as social network profiles (types of relationships, types of sexual and/or social networks across migration backgrounds, etc) and link these with SNT outcomes. Factors associated with (un)successful distribution of HIV self-tests will be examined by stepwise (logistical) regression analyses.

Usability of the e-learning tool for peers will be evaluated by using mixed methods approaches (qualitative interviews and a follow-up questionnaire) to gain insight on its user friendliness, record characteristics of peers (not) finishing the e-tool, and support enhancements in the (re)usability of the e-learning tool. Quantitative data will be analyzed using SAS/STAT version 14.1 (SAS Institute Inc) or SPSS Statistics version 24 (IBM Corp). Interview data will be transcribed using f4transcript version 7 (Audiotranskription) and assisted by NVivo software version 9 (QSR International) according to the thematic analysis method [29].

## Results

Between January and May 2019, 10 STI clinics and 7 HIV treatment clinics throughout the Netherlands (Amsterdam, Rotterdam, the Hague, Arnhem, Nijmegen, Utrecht, Flevoland, Groningen, Twente, and Brabant) began to recruit peers for the PREVENT pilot. A midterm and final evaluation will be conducted during 2019-2020.

## Discussion

### Aim

The PREVENT pilot is the first Dutch peer-moderated social network study using HIV self-tests and one of the first in Europe. This pilot intervention will provide insight on the feasibility and acceptability of SNT and its ability to identify undiagnosed HIV infections among MSM-NW in the Netherlands.

### Strengths and Limitations

A potential strength of this Web-based SNT approach is that hard-to-reach populations receive a free HIV self-test at a very low threshold, literally handed over by their friends. PREVENT has not only the ability to reach high-risk MSM-NW hidden to current HIV testing practices, it may also encourage conversations about risk behavior, HIV, and (barriers to) testing within the networks. Ultimately, the project can promote normalization of HIV testing. Also, the integrated online data collection, where peers and NAs complete questionnaires, enables anonymous linkage between the two groups. This provides us the opportunity to keep track of the distributed and used HIV self-tests.

There are also challenges to the success of our implementation. The value of SNT depends on many contextual factors as shown in previous SNT studies [10-13]. For SNT, it is important to include motivated peers who are willing to contribute to HIV testing in their community, an essential condition for optimal implementation of this study design. Therefore, peers who encounter challenges during the recruitment process will be personally assisted by the peer coordinator. Furthermore, our pilot focuses on a hard-to-reach population, and including 50 to 60 peers and 200 NAs in 12 to 18 months’ time is ambitious, especially due to the diversity of the target population in terms of cultural background, languages, educational levels, or socioeconomic statuses. Another concern is that the data collection on test results is more complicated when self-tests are used. Information on test results is provided by self-report in the follow-up questionnaire after 3 weeks and by collecting numbers of HIV confirmation tests conducted at the participating STI clinics. However, NAs might not be willing to report their test result. Also, NAs can go to their GP for a confirmation test or to an STI clinic outside the participating regions, which could lead to missing test results. Moreover, there is a possibility that NAs who accept the self-test will use the paper manual for instructions and not log in to the website. For them, epidemiological information and test results will also be missed. In addition, the evaluation process involves several small questionnaires that may be a barrier for NAs and peers. This could complicate a thorough evaluation of the outcomes of the pilot. Finally, language barriers are foreseen as the website and manuals are in three languages only. Peers could potentially bridge this barrier by helping NAs with the test and/or guiding them to the routine test locations. Hopefully, NAs who are tested for the first time through SNT find it easier to test themselves again in other settings (online or at STI clinics or GPs).

### Evaluation

During the evaluation of the pilot, we will focus on factors influencing all participation steps by peers and NAs, their feedback, and problem solving. We will also assess which peers reach the most NAs and who reaches NAs who have never tested before or tested positive. Factors include demographics, behavior, and process indicators. If needed, participation-enhancing strategies will be developed during the recruitment period. Creating general awareness for the pilot (ie, promotional material in the waiting room of the clinics) is already considered to stimulate engagement of peers in SNT. The first results may also support the need for further improvement of the content on the time2test website, the integrated e-tool for peers, or other elements of our pilot implementation. If SNT is acceptable and feasible among MSM-NW in the Netherlands, it can easily be adopted by other clinics and/or the website can be adjusted for future recruitment in other HIV risk populations.

## Appendix

Multimedia Appendix 1 Peer-reviewer report from Aidsfonds.

## Abbreviations

COM-B: capability, opportunity, motivation, and behavior
GP: general practitioner
LGBTI: lesbian, gay, bisexual, transgender, and intersex
MSM: men who have sex with men
MSM-NW: men who have sex with men–non-Western
NA: network associate
PrEP: pre-exposure prophylaxis
PREVENT: Peer-Empowered Voluntary Extended Network Testing
SNT: social network testing
STI: sexually transmitted infection
