# Supplementary material for: Web-Supported Social Network Testing for HIV Among Men Who Have Sex With Men With a Migration Background: Protocol for a Mixed Methods Pilot Study
Source: JMIR Res Protoc. 2020 Feb 10;9(2):e14743. doi: 10.2196/14743 (PMC7055807; doi:10.2196/14743)
Supplement: Multimedia Appendix 1 [file resprot_v9i2e14743_app1.pdf]

User:

**PROPOSAL NUMBER**	2016029 - III
Aids Fonds Conflict of Interest policy	Hereby, I declare that I am an independent referee, who has no conflict of interest with this proposal, according to the Aids Fonds Conflict of Interest policy. I will treat this proposal as confidential information.

Please note that the applicant will receive the complete review (anonymously). If you have any comments for Aids Fonds or the scientific advisory board, please send them to research@aidsfonds.nl

Below this review form you will find the application you are requested to review. You can open the application form in a new window and save as pdf by pressing "print".

1. Scientific quality

Importance of research question	3
Quality of research plan	3
Adequateness of research plan	3
Quality applicant	2
Expertise research group	2
Overall quality	3
To what extent is the proposed research plan suitable for answering the research questions? Please comment.	The research plan is well-designed to ask the research questions, focusing on evaluating the peer-driven Social Network Testing to improve HIV testing rates in the Netherlands.
Comment on the scientific quality	The proposal is limited in its impact on the initial seeds/peers (50-75 peers) and extended associates that would go for HIV testing (250-500 network associates, an average of 5 per peer). Without clear ascertainment of whether network associates undergoing further HIV test was purely influenced by their peer advice (i.e., not because of their routine HIV testing patterns, HIV/STI symptoms, doctors' advice etc.), it is very difficult to evaluate the overall success of such approach alone.

2. Innovativeness

Innovativeness of research question	3
Innovativeness of	3

approach	
Additional value to research already (being) performed in the field	3
Overall innovativeness	3
Comment on the innovativeness: what are the innovative aspects of the proposal? Will the research break new ground by generating new concepts, a deeper understanding, new methods for the HIV-field?	Using peer social network to encourage HIV testing in the Netherland-this has never been applied before. However, this concept is not completely new in sexual health promotion, such as partner notification after HIV/STI diagnosis (which usually has a limited research duet to HIV-related stigma and discrimination in particular). The proposal has not explicitly discussed whether peer-initiated HIV testing is more likely to encourage their network associated to go to MSM friendly clinics or community-based HIV testing venues. Hence, it is unlikely that with such a broad stroke, such evaluation will generate ground breaking concepts or methods.

3. Originality

Originality of research question in the field	3
Additional value to research already (being) performed by the group	3
Overall originality	3
Comment on originality	The idea to trial and evaluate peer-driven HIV testing coupled with peer training and e-learning in the Netherland is sound. It is unclear to what extent social network concepts will be applied in this public health-focused approach.

4. Feasibility

Appropriateness of approach	3
Feasibility of approach	4
Feasibility of time schedule	3
Quality of research group	2
Facilities of research group	3
Please comment on the riskfactors (Q10d). Do you foresee severe consequences for the feasibility of the project?	As was done before with the Respondent Driven Sampling for behavioural surveillance amongst subpopulations at high risk of HIV, some studies were highly successfully if they engage with communities well whereas others fail spectacularly without properly addressing issues such as HIV status disclosure and stigma. The success of the proposed approach of using social network peers to encourage HIV testing is highly dependent on different dynamics of social networks and connections, of which was not clearly articulated in the proposal.
Can you identify additional riskfactors?	Whether the research team is able to engage communities that are not coming forward to HIV testing, including migrant/cultural and

	linguistically diverse MSM communities who may or may not identify as gay or homosexual.
Overall feasibility	4
Please comment on the feasibility of the proposal.	There is a considerable risk that this approach will work well among gay-identified MSM in urban areas who have already been tested previously. For those hard-to-reach MSM subpopulations where HIV is still seen as a taboo topic, peer-driven HIV testing may cause great distress to peers.

5. Requested support

Requested scientific personnel	2
Requested supporting personnel	2
Requested type of personnel	2
Requested support for materials	2
Use of animal model	4
Number of animals	4
Requested time (duration project)	2
Comment on requested support	Requested funding is adequate.

6. Relevance

**Scientific** relevance	4
Please comment on the scientific relevance and (possible) scientific impact.(Q9, 10b)	The proposal is of a medium quality in terms of scientific relevance and impact.
How well has the applicant incorporated patients and/or targetgroups in the preparation and the researchplans?	4
Please comment on the incorporation of patient and/or target groups in the proposal (Q10e). How could it be improved?	The team has worked with communities but the specific involvement of communities, apart from the importance of selecting peers, was not mentioned.
**Societal** relevance	4
Please comment on the societal relevance and the (possible) societal impact. (Q9a,b,e)	The impact on hard-to-reach MSM who have never tested for HIV is likely to be limited.

How well has the applicant thought through the dissemination of the results. Can you identify any other opportunities?	The community dissemination was not mentioned.

Please answer the following questions as elaborate as needed. If insufficient argumentation is provided, we will unfortunately not be able to use your review.

**7. Could you please summarize or briefly comment** (point by point) **on the strengths of the proposed research?**	1. applying social network concepts and using peers to encourage HIV testing among MSM in the Netherlands, which has not been done before<br>2. conducting a comprehensive evaluation of the peer-driven HIV testing approach<br>3. combining project evaluation with online health promotion
**8. Could you please summarize or briefly comment** (point by point) **on the weaknesses of the proposed research?**	1. not able to precisely identify outcomes (how many HIV testing from network associated were indeed driven by peer influence)<br>2. not adequately unpack dynamics and heterogeneity of various social networks and connections<br>3. not actively involve and collaborate with community organisations and representatives from a range of background to ensure the success of such approach

9. Excellence

The Aids Fonds Netherlands has the resources to honor 10-15% of the submitted proposals. The subjects range from social sciences to clinical studies, from epidemiological studies to basic science.
(Find information about past funded projects here)

With this information in mind, would you place this proposal in the top 15%?	No
Please explain your answer.	The proposal is of a medium quality in terms of scientific quality and impact.

10. Fundability

Could you please classify the overall fundability of the proposed research according to the categories below?

Overall fundability of the proposal	4

Excellent: The applicant and research proposal are of the highest quality. The proposed research is at the forefront internationally, and will have substantial and innovative impact. Funding is highly recommended.
Very good:The applicant and/or research proposal are of a high quality. The proposed research is internationally competitive and will make a significant contribution. Funding is recommended.
Good: The applicant and/or research proposal are of a good quality. The proposed research will make a valuable contribution, but has some minor weaknesses. Funding is recommended only if ample resources are available.
Average: The applicant and/or research proposal are of sufficient quality. The proposed research will provide some new insights, but has significant weaknesses. Funding of the proposal in its present form is not recommended.
Poor: Unsuccessful in this form. The applicant and/or research proposal lack sufficient quality. The proposed research is weak in its scientific and/or methodological approach, and/or repeats other work. Funding is not recommended.

Leave here any additional comments/questions you may have for the applicants.

Aids Fonds

User:

**PROPOSAL NUMBER**	2016029 - II
Aids Fonds Conflict of Interest policy	Hereby, I declare that I am an independent referee, who has no conflict of interest with this proposal, according to the Aids Fonds Conflict of Interest policy. I will treat this proposal as confidential information.

Please note that the applicant will receive the complete review (anonymously). If you have any comments for Aids Fonds or the scientific advisory board, please send them to research@aidsfonds.nl

Below this review form you will find the application you are requested to review. You can open the application form in a new window and save as pdf by pressing "print".

1. Scientific quality

Importance of research question	2
Quality of research plan	2
Adequateness of research plan	3
Quality applicant	2
Expertise research group	1
Overall quality	2
To what extent is the proposed research plan suitable for answering the research questions? Please comment.	This is a proposed pilot study using an approach tested within another area of Europe and previously untried in The Netherlands to target hard to reach MSM who have not previously tested for HIV or have not recently tested. The research questions are centred around whether the use of Peer distributors of HIV home testing kits increases testing and can reach those that need to test within the peer educators social network. The proposed methodology goes some way in answering these questions adequately.
Comment on the scientific quality	this is a pilot study however a study like this would not be able to ascertain increase in uptake of testing (there is no denominator), and whether people would test elsewhere. this study would however provide some useful data on whether it was feasible to distribute kits via social networks, potential barriers and facilitators and whether the 'right kind' of Peers could be recruited to distribute to hard to reach groups of MSM, including those needing to test, not previously tested and those from other high risk MSM groups outside the culture of 'gay men'. i.e. immigrant groups and non-identifying gay bisexual men. I think the use of the oraquick is problematic for the research study as there is great potential for attrition of data and not knowing whether the kits were used, by whom, and what the results were.

2. Innovativeness

Innovativeness of research question	3
Innovativeness of approach	2
Additional value to research already (being) performed in the field	2
Overall innovativeness	2
Comment on the innovativeness: what are the innovative aspects of the proposal? Will the research break new ground by generating new concepts, a deeper understanding, new methods for the HIV-	A previously tested approach of Social Network Testing, not previously applied in the Netherlands which is its novel factor. Potentially could yield some interesting data. Identifying and targeting hard to reach MSM is the holy grail of reducing undiagnosed infection. This proposal offers one approach that may prove useful. This is a pilot study but will answer questions as to the feasibility and acceptability of using SNT and to a lesser degree may indicate whether it could potentially reach those who need to test. it may offer some insight into why people do not test - with careful data

field?	capture using the Peers.

3. Originality

Originality of research question in the field	2
Additional value to research already (being) performed by the group	2
Overall originality	2
Comment on originality	Seems original and novel as above.

4. Feasibility

Appropriateness of approach	3
Feasibility of approach	3
Feasibility of time schedule	2
Quality of research group	2
Facilities of research group	2
Please comment on the riskfactors (Q10d). Do you foresee severe consequences for the feasibility of the project?	Targeting hard to reach high risk MSM who do not test or do not test regularly is one of the primary on-going areas of further research. There are many reasons why men do not test and understanding this would hopefully open up avenues for intervention. This proposal goes some way to providing a new and previously untried method in the Netherlands for disseminating kits to 'potentially' hard to reach MSM. However, the use of HIV test kits over sampling kits may be problematic for a number of reasons. 1) will not be able to gauge whether the kit is used or not used, or who it is used by and what the result was. There is over reliance for these important outcomes to be collected by voluntary login by NA. secondly, there is a missed opportunity to link into care, unlike the use of HIV self sampling kits which would provide a more accurate picture of uptake and test result, whilst also being able to follow-up the person for other outcomes. The risk is that there will be a large loss to follow-up using the suggested approach and the missed opportunity to link these hard to reach people into care which is a crucial step in treatment and prevention.
Can you identify additional riskfactors?	The risk, without careful selection and recruitment of peers, is that hard to reach groups will not be targeted. it is not clear whether peers should fulfil some criteria for entry into the study. I appreciate some of this detail will be ascertained with the preliminary focus groups, however, a sampling grid could be applied at this stage to maximise demographic network spread for this pilot, i.e. age, risk behaviours, social network, ethnicity, urban vs rural, socio-demographic status etc.<br><br>it may also be more cost-effective to recruit less peers to distribute more kits, again knowing more about social network sizes would

	assist with estimating range of kits distributed.<br><br>The proposal indicates that peers will be eligible and selected if they 'highly motivated' and have high levels of 'self-efficacy'. more detail is needed as to how this will be achieved using the e-tool, whether this tool has already been developed or whether this is part of the project (is this adequately funded?) and how motivation and self-efficacy will be operationalized within the project.<br><br>Although the pilot study will go some way to ascertain this, it may be highly likely that highly motivated peers will not have the appropriate social networks and thus will not target hard to reach non-testers.
Overall feasibility	3
Please comment on the feasibility of the proposal.	I think the key to the whole project centres around its ability to recruit the right kind of peers and to ensure a good distribution at the outset. Use of a sampling grid may provide guidance for this. there is little detail on how high levels of motivation and self-efficacy will be ensured, what the cut-offs would be, how assessed and what would happen to those peers who fall short of this? will they be retrained or just rejected from the study?

5. Requested support

Requested scientific personnel	1
Requested supporting personnel	1
Requested type of personnel	1
Requested support for materials	1
Use of animal model	4
Number of animals	4
Requested time (duration project)	1
Comment on requested support	appropriate, although I would have liked to have seen less peers involved, distributing more test kits.

6. Relevance

**Scientific** relevance	3
Please comment on the scientific relevance and (possible) scientific impact.(Q9, 10b)	this is a pilot proposal but could potentially have relevance for a larger trial if SNT methods proved acceptable, feasible and showed potential reach to those undertested.
How well has the applicant incorporated patients and/or targetgroups in the preparation and the	3

researchplans?

Please comment on the incorporation of patient and/or target groups in the proposal (Q10e). How could it be improved?	MSM are used in the study, not sure how involved they were formulating the research questions or as collaborators rather than research subjects.
**Societal** relevance	2
Please comment on the societal relevance and the (possible) societal impact. (Q9a,b,e)	MSM are one of the highest risk groups for HIV in Europe. reducing undiagnosed infection remains key to preventing onward transmission.
How well has the applicant thought through the dissemination of the results. Can you identify any other opportunities?	fine, European conferences could be incorporated and would be relevant.

Please answer the following questions as elaborate as needed. If insufficient argumentation is provided, we will unfortunately not be able to use your review.

**7. Could you please summarize or briefly comment** (point by point) **on the strengths of the proposed research?**	The strengths are the research team, the use of a novel approach to the problem of identifying and targeting testing at previously untested or undertested groups of MSM, the SNT approach. The use of social science techniques to tackle the problem of undiagnosed infection which are needed.
**8. Could you please summarize or briefly comment** (point by point) **on the weaknesses of the proposed research?**	The use of the self-testing kits rather than use of self-sampling kits, the lack of concrete questions around acceptability and feasibility - what are indicators of success for this pilot? The lack of a use of a sampling grid or similar to guide recruiting a breadth of Peers to the study.

9. Excellence

The Aids Fonds Netherlands has the resources to honor 10-15% of the submitted proposals. The subjects range from social sciences to clinical studies, from epidemiological studies to basic science. (Find information about past funded projects here)

With this information in mind, would you place this proposal in the top 15%?	Yes
Please explain your answer.	Social science research is much needed in this area where the onus is understanding why people do not test and how to potentially identify and reach them. This study would add good data to this under researched area.

10. Fundability

Could you please classify the overall fundability of the proposed research according to the categories below?

Overall fundability of the	2

proposal

Excellent: The applicant and research proposal are of the highest quality. The proposed research is at the forefront internationally, and will have substantial and innovative impact. Funding is highly recommended.
Very good:The applicant and/or research proposal are of a high quality. The proposed research is internationally competitive and will make a significant contribution. Funding is recommended.
Good: The applicant and/or research proposal are of a good quality. The proposed research will make a valuable contribution, but has some minor weaknesses. Funding is recommended only if ample resources are available.
Average: The applicant and/or research proposal are of sufficient quality. The proposed research will provide some new insights, but has significant weaknesses. Funding of the proposal in its present form is not recommended.
Poor: Unsuccessful in this form. The applicant and/or research proposal lack sufficient quality. The proposed research is weak in its scientific and/or methodological approach, and/or repeats other work. Funding is not recommended.

Leave here any additional comments/questions you may have for the applicants.	Its challenging to assess this application without looking at the standard across the scheme. However, this team are very well placed to carry out this research and this pilot could provide useful data to support the armoury of different approaches to target hard to reach MSM. there has been little that has been successful to date and therefore I support novel approaches. I think the use of oraquick is potentially missing a trick to provide linkage into care and reduce the amount of missing data that I suspect could be large in a study like this. If there was scope to revise elements of the study then it would make a bigger impact to our knowledge.

Aids Fonds

User:

**PROPOSAL NUMBER**	2016029 - I
Aids Fonds Conflict of Interest policy	Hereby, I declare that I am an independent referee, who has no conflict of interest with this proposal, according to the Aids Fonds Conflict of Interest policy. I will treat this proposal as confidential information.

Please note that the applicant will receive the complete review (anonymously). If you have any comments for Aids Fonds or the scientific advisory board, please send them to research@aidsfonds.nl

Below this review form you will find the application you are requested to review. You can open the application form in a new window and save as pdf by pressing "print".

1. Scientific quality

Importance of research question	2
Quality of research plan	2
Adequateness of research plan	3
Quality applicant	2
Expertise research group	1
Overall quality	2
To what extent is the proposed research plan suitable for answering the research questions? Please comment.	I think this is a good study design, assessing a method of getting HIV tests to hard-to-reach individuals.<br><br>Some attempt should be made to assess cost-effectiveness if possible. Modelling may be required.<br><br>Frequency of repeat testing would also be a useful outcome.
Comment on the scientific quality	After reading the outcome measures I'm not sure how you would decide if the method was ineffective. I didn't see the sample size calculation if there was one. Is the sample size large enough to ensure the research question is answered, or is it larger than needed?

2. Innovativeness

Innovativeness of research question	1
Innovativeness of approach	1
Additional value to research already (being) performed in the field	1
Overall innovativeness	1
Comment on the innovativeness: what are the innovative aspects of the proposal? Will the research break new ground by generating new concepts, a deeper understanding, new methods for the HIV-field?	Using peer-distributed home HIV tests to unearth undiagnosed cases of HIV is novel and has not been previously studied as far as I know. There is a study of home HIV testing in Australia, looking at frequency of testing, but this is a different outcome.

3. Originality

Originality of research question in the field	1
Additional value to research already (being) performed by the group	2
Overall originality	1
Comment on originality	I've not seen a study like this before.

4. Feasibility

Appropriateness of approach	2
Feasibility of approach	2
Feasibility of time schedule	2
Quality of research group	1
Facilities of research group	2
Please comment on the riskfactors (Q10d). Do you foresee severe consequences for the feasibility of the project?	No. I think the home tests will be popular or they won't be, and that's what the project aims to measure.
Can you identify additional riskfactors?	Oral HIV tests have slightly longer window periods than other rapid tests and significantly longer than laboratory based 4th generation HIV tests (median 3 weeks in one study - Masciotra J Clin Virology 2011). People with acute infection who have negative oral tests may transmit infection in the belief that they are HIV negative. The oral test could arguably provide false reassurance. I'm not sure that this should stop the study, but the study team needs to address this risk.
Overall feasibility	2
Please comment on the feasibility of the proposal.	I think the popularity of home tests will likely make this a successful initiative in terms of test uptake. It will be harder to determine whether individuals are reached who might have been reached by less expensive clinic-based testing and therefore harder to determine cost-effectiveness.

5. Requested support

Requested scientific personnel	1
Requested supporting personnel	1
Requested type of personnel	2
Requested support for	1

materials	
Use of animal model	3
Number of animals	3
Requested time (duration project)	1
Comment on requested support	Seems realistic.

6. Relevance

**Scientific** relevance	2
Please comment on the scientific relevance and (possible) scientific impact.(Q9, 10b)	There is a great need to attract gay men to test for HIV frequently. Frequency of repeat testing and the number of cases diagnosed per test, will be important outcomes.
How well has the applicant incorporated patients and/or targetgroups in the preparation and the researchplans?	2
Please comment on the incorporation of patient and/or target groups in the proposal (Q10e). How could it be improved?	Hard to see how it could be improved. But worth measuring to what extent this intervention simply replaces going to the clinic for a test, versus encouraging additional testing.
**Societal** relevance	2
Please comment on the societal relevance and the (possible) societal impact. (Q9a,b,e)	Highly relevant.
How well has the applicant thought through the dissemination of the results. Can you identify any other opportunities?	This is fine.

Please answer the following questions as elaborate as needed. If insufficient argumentation is provided, we will unfortunately not be able to use your review.

**7. Could you please summarize or briefly comment** (point by point) **on the strengths of the proposed research?**	Novel research question and method of test distribution.<br><br>Socially worthwhile goal of reaching undiagnosed HIV now that we know that treatment prevents infection.
**8. Could you please summarize or briefly comment** (point by point) **on the weaknesses of the proposed research?**	Further detail would help on:<br>- the impact of the longer window period of the oral test (but for some patients, any test is better than none, so this is not a fatal flaw)<br>- frequency of testing<br>- cost-effectiveness of the intervention<br>- impact of the intervention on attendance for clinic-based testing

- sample size

9. Excellence

The Aids Fonds Netherlands has the resources to honor 10-15% of the submitted proposals. The subjects range from social sciences to clinical studies, from epidemiological studies to basic science.
(Find information about past funded projects here)

With this information in mind, would you place this proposal in the top 15%?	Yes
Please explain your answer.	If this method proves effective in reaching a quantifiable proportion of undiagnosed HIV, or leading to measurably early diagnosis, then yes, it's in the top bracket. I think the above questions require more detail to be sure about this. A control intervention would be worth considering. I have scored the "fundability" as good. I would rate it higher if there was more detail provided on the issues I have raised.

10. Fundability

Could you please classify the overall fundability of the proposed research according to the categories below?

Overall fundability of the proposal	3

Excellent: The applicant and research proposal are of the highest quality. The proposed research is at the forefront internationally, and will have substantial and innovative impact. Funding is highly recommended.
Very good:The applicant and/or research proposal are of a high quality. The proposed research is internationally competitive and will make a significant contribution. Funding is recommended.
Good: The applicant and/or research proposal are of a good quality. The proposed research will make a valuable contribution, but has some minor weaknesses. Funding is recommended only if ample resources are available.
Average: The applicant and/or research proposal are of sufficient quality. The proposed research will provide some new insights, but has significant weaknesses. Funding of the proposal in its present form is not recommended.
Poor: Unsuccessful in this form. The applicant and/or research proposal lack sufficient quality. The proposed research is weak in its scientific and/or methodological approach, and/or repeats other work. Funding is not recommended.

Leave here any additional comments/questions you may have for the applicants.
